# Assessment of respiratory muscle strength in children according to the classification of body mass index

**DOI:** 10.1590/0103-0582201432210313

**Published:** 2014-06

**Authors:** George Jung da Rosa, Camila Isabel S. Schivinski

**Affiliations:** 1 Udesc, Florianópolis, SC, Brasil

**Keywords:** muscle strength, respiratory muscles, child, body mass index

## Abstract

**OBJECTIVE:**

To assess and compare the respiratory muscle strength among eutrophic, overweight and obese school children, as well as to identify anthropometric and respiratory variables related to the results.

**METHODS:**

Cross-sectional survey with healthy schoolchildren aged 7-9 years old, divided into three groups: Normal weight, Overweight and Obese. The International Study of Asthma and Allergies in Childhood (ISAAC) questionnaire was applied. The body mass index (BMI) was evaluated, as well as the forced expiratory volume in one second (FEV_1_) with a portable digital device. The maximal inspiratory and expiratory pressures (MIP and MEP) were measured by a digital manometer. Comparisons between the groups were made by Kruskal-Wallis test. Spearman's correlation coefficient was used to analyze the correlations among the variables.

**RESULTS:**

MIP of eutrophic school children was higher than MIP found in overweight (*p*=0.043) and obese (*p*=0.013) children. MIP was correlated with BMI percentile and weight classification (r=-0.214 and r=-0.256) and MEP was correlated with height (r=0.328). Both pressures showed strong correlation with each other in all analyses (r≥0.773), and less correlation with FEV_1_ (MIP - r=0.362 and MEP - r=0.494). FEV_1_ correlated with MEP in all groups (r: 0.429 - 0.569) and with MIP in Obese Group (r=0.565). Age was correlated with FEV_1_ (r=0.578), MIP (r=0.281) and MEP (r=0.328).

**CONCLUSIONS:**

Overweight and obese children showed lower MIP values, compared to eutrophic ones. The findings point to the influence of anthropometric variables on respiratory muscle strength in children.

## Abstract (es)

**OBJETIVO:**

Evaluar y comparar la fuerza muscular respiratoria de escolares eutróficos, con sobrepeso y obesos, así como identificar variables antropométricas y respiratorias que se relacionen con los resultados.

**MÉTODOS:**

Estudio transversal con escolares sanos de 7 a 9 años, divididos en tres grupos: Eutróficos, Sobrepeso y Obesos. Se aplicó el cuestionario del *International Study of Asthma and Allergies in Childhood *(ISAAC) y se evaluaron el índice de masa corporal (IMC), el volumen espiratorio forzado en el primero segundo (VEF_1_), mediante una lectora digital portátil, y las presiones inspiratorias y espiratorias máximas (PIMáx y PEMáx), medidas por manovacuometría digital. Se compararon los grupos por la prueba de Krulkal-Wallis, seguida del análisis por la prueba de Mann-Whitney cuando se constató diferencia significativa. Se aplicó el coeficiente de correlación de Spearman para analizar correlaciones entre las variables.

**RESULTADOS:**

la PIMáx de escolares eutróficos fue más grande que la de los con sobrepeso (*p*=0,043) y la de los obesos (*p*=0,013). La PIMáx se correlacionó con el percentil y la clasificación según el IMC (r=-0,214 y r=-0,256) y la PEMáx, con la estatura (r=0,328). Ambas presiones mostraron fuerte correlación entre sí en todos los análisis (r≥0,773) y débil correlación con VEF_1_ (PIMáx - r=0,0362 y PEMáx - r=0,494). El VEF_1_ se correlacionó con la PEMáx en los tres grupos (r de 0,429 a 0,569) y con la PIMáx en el Grupo Obeso (r=0,565). La edad presentó relación con las variables VEF_1_ (r=0,578), PIMáx (r=0,281) y PEMáx (r=0,328).

**CONCLUSIONES:**

Escolares obesos y con sobrepeso presentaron valores inferiores de PIMáx en comparación a los eutróficos. Los hallazgos apuntan a la influencia de variables antropométricas en la fuerza muscular respiratoria en niños.

## Introduction

Excess weight is a public health problem that burdens public coffers in more than R$ 488 million annually. The proportion of overweight Brazilians increased from 42.6% in 2006 to 48.5% in 2011, while the percentage of obese rose from 11.4 to 15.8% in the same period^(^ 1 ^)^. According to the Brazilian Institute of Geography and Statistics (Instituto Brasileiro de Geografia e Estatística - IBGE), one in every three children from 5-9 is overweight and, in the age range from 10-19 years, the index reaches 21.7%, which represents a 7-fold increase in the last 3 decades^(^ 2 ^)^.

The monitoring of obesity shows that 80% of obese children will be obese adults, and retrospective studies show that 30% of obese adults were obese children^(^ 3 ^)^. Among the complications associated with obesity, the following stand out: hypertension, diabetes, psychosocial disorders related to acceptance in the group, and removal of group activities, sleep apnea, and increased ventilatory demand^(^ 4 ^)^. This increased ventilatory demand is often accompanied by fatigue upon exertion and limitations to carry out some activities of daily living.

Also from the respiratory point of view, obese people may present changes in the distribution of ventilation, with the risk of manifesting gas exchange abnormalities. Commonly, there is a reduction in spirometric variables of functional residual capacity and expiratory reserve volume due to the presence of accumulated adipose tissue around the thoracic and abdominal surfaces^(^ 5 ^-^ 7 ^)^. With the deposition of fat in these compartments, pulmonary compliance can be reduced by up to 66%, implying damage to mechanical ventilation with increasing respiratory effort, potential inefficiency, and decreased ability to generate strength for ventilation. 

In this line, the relationship between obesity and respiratory muscular strength (RMS) has been studied, but without conclusive results^(^ 7 ^-^ 9 ^)^, especially in children, in which studies are still limited. In this context, the objective of this study was to assess and compare the RMS by means of maximum respiratory pressures in eutrophic, overweight, and obese school children, and to identify anthropometric and respiratory variables that are related to the results found.

## Method

Cross-sectional study performed in the schools within the municipality of Florianópolis, state of Santa Catarina, in the period from February to April 2013. Three educational institutions agreed to participate, two private and one public. The sample was chosen by convenience and consisted of children aged from 7-9 years of both sexes. Inclusion criteria were healthiness and the ability to understand and properly perform the tests involved in the research.

Children's healthiness was demonstrated through The International Study of Asthma and Allergies in Childhood (ISAAC) questionnaire, administered to parents. This protocol is a respiratory symptoms questionnaire used to assess the prevalence of asthma, rhinitis, and eczema for the past 12 months. The following modules were applied: 1) asthma^(^ 10 ^)^, which included wheezing-related issues: frequency, trigering factors, and severity, in addition to the previous diagnosis of the disease; 2) rhinitis^(^ 11 ^)^, with explanation about the occurrence, frequency, and intensity of sneezing and runny nose, apart from previous medical diagnosis of the disease. Children with asthma module score ≤5 and rhinitis module ≤4 were considered healthy. A history of children's health prepared by the researchers was also applied, consisting of questions concerning physical activity, medications, existing or preterit diagnosed diseases and hospitalizations, to confirm the healthiness. 

We excluded children with a history of cardiorespiratory, neuromuscular, rheumatic, and neurological diseases and those with any acute illness at the time of collection or impossibility of performing assessment procedures properly. We also excluded students whose health questionnaire was answered with dubious content on the child's he healthiness and those with forced expiratory volume in one second (FEV1) lower than 80% of the predicted, according to Polgar and Weng^(^ 12 ^)^. 

After obtaining the schools' consent regarding participation, we conducted the collection at the School, always by the same evaluator, in a reserved place to conduct the procedures. All participants received and returned the term of consent signed by parents or legal guardians. The study (CAAE n. 01821712.6.0000.0118) was approved by the Research Ethics Committee of Universidade do Estado de Santa Catarina under n. 63455. 

Initially, we assessed body weight (0.1kg accuracy) and height (0.5cm accuracy) using a stadiometer (Welmy 200/5). Anthropometric measurements were conducted with the child remaining with the body erect and aligned, with heels, calves, buttocks, shoulder blades, and occiput touching the stadiometer. At the time of evaluation, the participants wore school uniform shirts, shorts or pants and were barefoot. Subsequently, the values ​​previously obtained for shirts (150g), shorts (150g), or pants (250g) were subtracted from the measured value of mass. 

Once the values ​​of body weight and height were obtained, we calculated body mass index (BMI) with the BMI Child calculator by the Brazilian Ministry of Health^(^ 13 ^)^. It is an instrument where you enter data on weight, height, sex, and age of the child. Once processed, the calculator obtains the value of BMI, the percentile, and, from this, the diagnosis of tropism. Based on this information, children were gathered into three groups, determined by the percentile in which the child was in the BMI/age curve, namely: Eutrophic Group (EG - for those belonging to percentiles greater than 3 and lower than 85); Overweight Group (OG - for percentiles equal to or greater than 85 and equal to or less than 97); and Obese Group (ObG - when percentiles were greater than 97)^(^ 13 ^)^.

After anthropometry, the same examiner performed the measurement of FEV1, measured with a digital monitor (Piko-1, Spire Health, USA). The measurements were taken according to the standards and criteria of respiratory muscle function declaration for the American Thoracic Society (ATS) and the European Respiratory Society (ERS)^(^ 14 ^)^ with the child sitting, back against the back of the chair, head aligned, and upper limbs rested on the bottom. We used a nose clip and the child was asked to perform a maximal inspiration followed by forced expiration, with verbal stimuli. We recorded the highest value of three measurements with an interval of 30 seconds between them, two of which should not differ by more than 0.15 L, in a maximum of five maneuvers. In case we did not obtain acceptable measurements, the test was disregarded.

Then the RMS was verified using a digital manometer with one-way valve (MVD300, G-MED, Brazil). The measurement system has a 2mm-diamter hole to prevent glottic closure during the maneuver of maximal inspiratory pressure (MIP) and reduce the use of buccal muscles during the maximal expiratory pressure (MEP) maneuver. Following guidelines and demonstrations on the test, the examiner offered verbal encouragement for the child to perform a maximal inspiration followed by a maximal expiration through a nozzle held tightly around the lips to prevent leaks. During the test, the student sat with his/her back on the chair, feet on the floor, upper limbs resting on the bottom, and made use of a nose clip. Measurements were performed according to the standards and criteria of the declaration of the ATS for respiratory muscle function^(^ 15 ^)^. To obtain the MIP, the child expired until the next residual volume and then performed a maximal inspiration. The MEP was measured from a breath with almost total pulmonary capacity, followed by a maximal expiration. There were at least three and a maximum of seven maneuvers for each of the measures of MIP and MEP. If the measurements obtained were not acceptable and reproducible, the test was considered invalid. We considered satisfactory measures when the maximum value of three acceptable (no leaks and lasting at least about 2 seconds) and reproducible maneuvers varied less than 20% between each other, being recorded the greatest measure. For each maneuver of each measure, there was an interval of 30 to 40 seconds^(^ 15 ^)^. Between the measurement of MIP and MEP, there was an interval of 3 minutes to avoid fatigue. 

To calculate the sample size, we considered the result of the MIP from a pilot study in which it presented a standard deviation of 10cmH_2_O. To detect a difference of 5cmH_2_O and a test power of 80%, with significance level of 5%, 25 schoolchildren were estimated in each group^(^ 16 ^)^. 

For data analysis, the numerical parameters were imported into Microsoft Excel^(r)^ 2010 and, subsequently, transferred to the Windows Statistical Package for Social Sciences (SPSS) 20.0 for statistical processing. Initially, we used descriptive and frequency statistics, with data expressed as mean and standard deviation. We applied the Kolmogorov-Smirnov normality test and then, to compare the three groups, we used the non-parametric Kruskal-Wallis test. Once the difference between groups was identified, we used the Mann-Whitney test to find differences by comparing two groups at a time. To identify correlations between variables, we applied the Spearman correlation. The level of significance was established at 0.05.

## Results

Adding the three institutions involved, 112 schoolchildren were analyzed and 90 were part of the sample, 30 in each of the groups, with 15 of each sex and ages from 7 to 9. Of the total number of children assessed, 16 were excluded due to chronic or acute disease, four by presenting less than 80% of the predicted VEF_1_, and two for not completing the required tests. Sample characterization of each of the three groups, according to the anthropometric data of weight, height, BMI, and percentile, and the respiratory variables MIP, MEP, and VEF_1 _are shown in Table 1. 

**Table 1:**
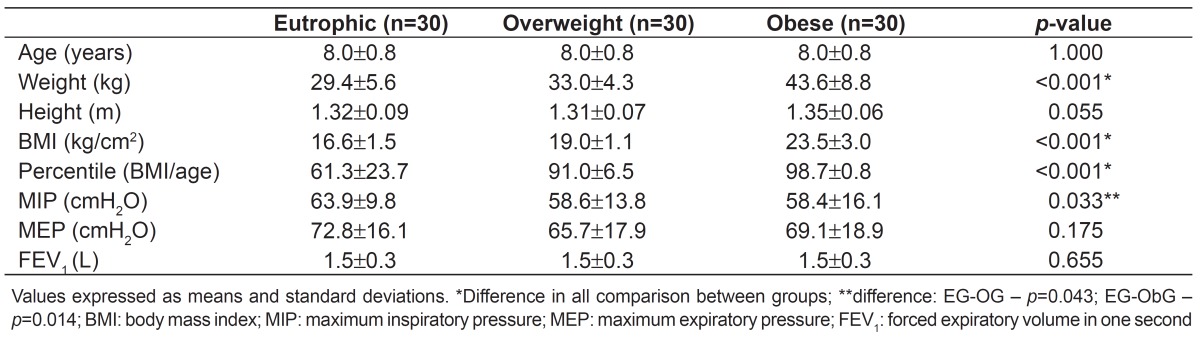
Characteristics of the sample according to the anthropometric and respiratory variables and results of the comparison of the data by the Kruskal-Wallis test

As already expected given the preliminary characterization of groups in EG, OG, and ObG by BMI, we found significant differences in the anthropometric variables of weight (*p*<0.001), BMI (*p*<0.001), and percentile (*p*<0.001), in the comparison between groups using the Kruskal-Wallis test. Among the respiratory variables, the groups differed only in MIP (p=0.033). The analysis between two groups at a time by using the Mann-Whitney test identified differences in MIP, with higher values ​​for the EG compared to the other groups (EG - 63.9±9.75 versus OG - 58.6±13.81, *p*=0.043; EG versus ObG - 58.36±16.14, *p*=0.013), with no significant difference between OG and ObG (58.6±13.81 *versus* 58.36±16, *p*=0.779). There was no difference between maximal respiratory pressures (MRP) by sex, in any of the three groups.

Spearman correlation was applied between the data of the variables in the total sample, and we observed that age correlated with the variables VEF_1_ (r=0.578), MIP (r=0.281), and MEP (r=0.328). There was also negative correlation with the percentile and the classification according to BMI (BMIclass) (r=-0.214 and r=-0.256). The MRPs strongly correlated with each other (r=0.822) and less intensively with VEF_1_ (MIP, r=0.362 and MEP, r=0.494). The MEP presented relation to height (r=0.328) and to VEF_1_ (r=0.488).

In the analysis of each group, age presented values of correlation, except for the OG, with mass (EG - r=0.722; ObG - r=0.380) and height (EG - r=0.772; ObG - r=0.513). Mass was also correlated with height, as expected (EG - r=0.895; OG - r=0.910; ObG - r=0.843) and with BMI (EG - r=0.848; OG - r=0.530; ObG - r=0.896). The MIP was correlated with age and height only in EG (r=0.389), with the VEF_1_ in ObG (r=0.565) and with MEP in all groups (EG - r=0.773; OG - r=0.795; ObG - r=0.910). The MEP was also correlated to age and height in the EG (r=0.413 and r=0.479, respectively) and with VEF_1_ in the three groups (EG - r=0.531; OG - 0.429; ObG - r=0.569). The VEF_1_, in turn, was correlated with age (EG - r=0.541; OG - r=0.663; ObG - r=0.438), mass (EG - r=0.438; OG - r=0.461), and height (EG - r=0.515; OG - r=0.379).

## Discussion

The respiratory muscles are responsible for generating pressure differences that ensure ventilation and, therefore, the measure of the RMS is considered indispensible and of great use in the evaluation of various states and diseases^(^ 17 ^)^. Among these clinical situations, concern about child obesity has been increasing. However, the implications of obesity on RMS are still not well defined, which motivated the present investigation.

Among the results, we identified a correlation between age and the MRPs both in the total sample and in the EG, which is in line with other studies^(^ 18 ^-^ 21 ^)^. It is interesting to note that the event was not repeated in the OG and the ObG, which may be an indicative of the involvement of MRPs in the presence of changes in body tropism, in case of overweight. This observation can be explained by the changes caused by the accumulation of fat in the thoracic and abdominal cavities, which can result in damages to the respiratory pump due to the functional alteration of the inspiratory and expiratory muscles^(^ 5 ^-^ 7 ^,^ 8 ^)^.

Age also correlated with FEV1 in all analyzes, and this spirometric variable showed close correlation with height. Such correspondence is justified by the proportionality of the body and respiratory growth in childhood, which occur with advancing age^(^ 22 ^)^. The literature has already described that event^(^ 12 ^,^ 23 ^-^ 25 ^)^, in which the height gain occurs according to age and the pulmonary function is characterized by the increased volumes following growth during childhood and adolescence. 

This linearity in somatic development can also support the strong correlation observed between MIP and MEP. In the case of this relationship between the MRPs, another point that maintains the correlation is the anatomical and functional contiguity between the thoracic and abdominal compartments. The inspiratory act, which, a priori, occurs in the thoracic compartment, occurs most effectively when the diaphragm finds abdominal muscles strong enough to give it support in the movement, effecting muscle synergism. In the sample of healthy children, it seems appropriate to note that the pressures grow together with age, giving the child appropriate inspiratory and expiratory responses in situations of increased ventilatory demand^(^ 26 ^)^.

Regarding the MIP, there was a decrease in the values ​​of EG, OG and ObG, supported by negative correlations - even if weak - of the variable with the percentile and also to the classification according to BMI. This finding may be explained by a dysfunction of the diaphragm related to the deposition of abdominal and visceral adipose tissue, leading to a disadvantage in the length-tension relationship, due to overstretching of the muscle fibers. The effects occur mainly in the inspiratory muscles, particularly the diaphragm, and the damage to the lung function worsened according to the degree of obesity^(^ 9 ^,^ 27 ^,^ 28 ^)^. Such arguments could also explain the negative correlation found between the MIP and the variables percentile and classification of tropism according to BMI, featuring the highest degree of overweight/obesity with greater trend to compromise the RMS. Corroborating this line, Santiago et al^(^ 8 ^)^ assessed children and adolescents grouped as overweight/obese, from 4 to 15 years, and found higher values of MEP in the eutrophic group (*p*=0.003). Researchers discuss a trend to decreased MIP for the overweight/obese (*p*=0.068) group, attributing the finding to abdominal fat distribution and its effect on the MRP. 

Still comparing RMS of obese and normal children, a Thai study^(^ 29 ^)^ found no significant differences between the groups, in the assessment of children from 10 to 12 years. According to Charususin et al^(^ 29 ^)^, adiposity did not interfere with the sample, since the participants had pulmonary function values within predicted. Another study^(^ 9 ^)^, which included older children and adolescents (from 9 to 17 years), showed no influence of body weight on the MRP. According to these authors, this finding may be due to the effect of constant training played by inspiratory overload imposed by the accumulation of adipose tissue.

Unlike this study, the aforementioned studies assessing the RMS according to tropism analyzed children and adolescents together, which may have influenced the results. This is because the body and ventilatory changes are significant after the transition of these two stages of life^(^ 12 ^,^ 24 ^,^ 26 ^)^. No studies were found with a similar methodology regarding the fact that participants were exclusively children aged from 7 to 9 years old, which did not allow further comparisons between the results. With regard to the absolute values ​​of MIP and MEP, when taken only children from the EG, it is observed that the values ​​reported in the literature are higher than those identified in this study. Domènech-Clar et al^(^ 20 ^)^ found in their sample, MIP of 79 and 68cmH_2_O and MEP of 95 and 82cmH_2_O for boys and girls from 8 to 10 years, respectively. Accordingly, higher values ​​are presented by Wilson^(^ 18 ^)^ and Szeinberg^(^ 19 ^)^, both also referenced by the American Thoracic Society^(^ 15 ^)^. This very publication^(^ 15 ^)^ draws attention to the need for regionalization of reference values and consequent caution in extrapolating the interpretation of results.

As demonstrated, the impact of obesity on ventilatory function has been often discussed, showing especially its effects on the MIP in the present investigation. Considering the increasing weight of children as a phenomenon of Public Health, which is continuously escalating, the study findings identify the importance of monitoring the RMS, especially in children with higher body mass indexes, to prevent possible respiratory disorders and complications. In this context, it is important to highlight a limitation of the present study regarding the inclusion of participants. The equipment used for the respiratory assessment of school children, Piko-1 (Spire Health, EUA), does not provide data for forced vital capacity (FVC), preventing the analysis of the relationship VEF_1_/FVC to exclude only children with obstructive respiratory disease.

We may conclude that obese and overweight children presented lower values ​​of MIP compared to eutrophic children. There was a strong relationship between MIP and MEP, being both related to age and FEV_1_, mainly in the Obese Group. MIP was associated with BMI, and MEP was associated with height, especially in the Eutrophic Group. The findings point to the influence of anthropometric variables on the RMS in children, as well as the relationship between strength and the spirometry parameter FEV_1_.
