# Supplementary material for: Assessment of respiratory muscle strength in children according to the classification of body mass index
Source: Rev Paul Pediatr. 2014 Jun;32(2):250–5. doi: 10.1590/0103-0582201432210313 (PMC4183012; doi:10.1590/0103-0582201432210313)
Supplement: Translation

# Translation: Avaliação da força muscular respiratória de crianças segundo a classificação do índice de massa corporal

**Authors:** George Jung da Rosa, Camila Isabel S. Schivinski

**Affiliations:** 1 Udesc, Florianópolis, SC, Brasil

**Keywords:** força muscular, músculos respiratórios, criança, índice de massa corporal

## Abstract

**OBJETIVO:**

Avaliar e comparar a força muscular respiratória de escolares eutróficos, com sobrepeso e obesos, bem como identificar variáveis antropométricas e respiratórias que se relacionem com os resultados.

**MÉTODOS:**

Estudo transversal com escolares hígidos de sete a nove anos, divididos em três grupos: Eutróficos, Sobrepeso e Obesos. Aplicou-se o questionário do *International Study of Asthma and Allergies in Childhood* (ISAAC) e avaliaram-se o índice de massa corpórea (IMC), o volume expiratório forçado no primeiro segundo (VEF_1_), por meio de um leitor digital portátil, e as pressões inspiratórias e expiratórias máximas (PIMáx e PEMáx), medidas por manovacuometria digital. Compararam-se os grupos pelo teste de Kruskal-Wallis. Aplicou-se o coeficiente de correlação de Spearman para analisar correlações entre as variáveis.

**RESULTADOS:**

A PIMáx de escolares eutróficos foi maior que a dos portadores de sobrepeso (*p*=0,043) e a dos obesos (*p*=0,013). A PIMáx correlacionou-se com o percentil e a classificação do IMC (r=-0,214 e r=-0,256) e a PEMáx, com a estatura (r=0,328). Ambas as pressões mostraram forte correlação entre si em todas as análises (r≥0,773) e fraca correlação com VEF_1_ (PIMáx - r=0,362 e PEMáx - r=0,494). O VEF_1_ correlacionou-se com a PEMáx nos três grupos (r=0,429-0,569) e com a PIMáx no Grupo Obeso (r=0,565). A idade apresentou relação com as variáveis VEF_1_ (r=0,578), PIMáx (r=0,281) e PEMáx (r=0,328).

**CONCLUSÕES:**

Escolares obesos e com sobrepeso apresentaram valores inferiores de PIMáx em comparação aos eutróficos. Os achados apontam para a influência de variáveis antropométricas na força muscular respiratória em crianças.

## Introdução

O excesso de peso é um problema de Saúde Pública que onera anualmente os cofres públicos em mais de R$ 488 milhões. A proporção de brasileiros com sobrepeso passou de 42,6% em 2006 para 48,5% em 2011, enquanto o percentual de obesos subiu de 11,4 para 15,8% no mesmo período^(^ ^1^ ^)^. Segundo o Instituto Brasileiro de Geografia e Estatística (IBGE), uma em cada três crianças de cinco e nove anos está acima do peso e, na faixa dos dez aos 19 anos, o índice chega a 21,7%, o que representa crescimento de sete vezes nas últimas três décadas^(^ ^2^ ^)^.

O acompanhamento da obesidade mostra que 80% das crianças obesas serão adultos obesos e estudos retrospectivos demonstram que 30% dos adultos obesos foram crianças obesas^(^ ^3^ ^)^. Dentre as complicações associadas à obesidade, destacam-se: hipertensão arterial, diabetes, distúrbios psicossociais relacionados à aceitação no grupo e afastamento das atividades em grupo, apneia do sono e aumento da demanda ventilatória^(^ ^4^ ^)^. Esse aumento da demanda ventilatória, muitas vezes, é acompanhado de fadiga aos esforços e limitações para algumas atividades de vida diária.

Também do ponto de vista respiratório, pessoas obesas podem apresentar alterações na distribuição da ventilação, com risco de manifestar anomalias nas trocas gasosas. Comumente, ocorre redução das variáveis espirométricas de capacidade residual funcional e volume de reserva expiratório devido à presença de tecido adiposo acumulado ao redor das superfícies torácica e abdominal^(^ ^5^ ^-^ ^7^ ^)^. Com a deposição de gordura nesses compartimentos, a complacência pulmonar pode ser reduzida em até 66%, levando a prejuízos à mecânica ventilatória, com aumento do trabalho respiratório, eventual ineficiência e diminuição da capacidade de gerar força para a ventilação. 

Nessa linha, a relação entre a obesidade e a força muscular respiratória (FMR) tem sido estudada, porém sem resultados conclusivos^(^ ^7^ ^-^ ^9^ ^)^, sobretudo em crianças, nas quais os estudos ainda são escassos. O objetivo deste estudo foi avaliar e comparar a FMR por meio das pressões respiratórias máximas em escolares eutróficos, com sobrepeso e obesos, e identificar variáveis antropométricas e respiratórias que se relacionem com os resultados encontrados.

## Método

Estudo transversal realizado na rede escolar dos municípios da região de Florianópolis, SC, no período de fevereiro a abril de 2013. Consentiram com a investigação três instituições de ensino, duas privadas e uma pública. A amostra foi do tipo conveniência e constituída de crianças de sete a nove anos de idade de ambos os sexos. Os critérios de inclusão consistiram na higidez e na capacidade de compreender e executar adequadamente os testes envolvidos na pesquisa.

Comprovou-se a higidez respiratória das crianças por meio do questionário *The International Study of Asthma and Allergies in Childhood* (ISAAC), aplicado aos pais. Esse protocolo é um questionário de sintomas respiratórios utilizado para avaliar a prevalência de asma, rinite e eczema com questões relativas aos últimos 12 meses. Aplicaram-se os módulos: 1) asma^(^ ^10^ ^)^, no qual se apresentaram questões relacionadas à sibilância: frequência, fatores desencadeantes e gravidade, além do diagnóstico prévio da doença; 2) rinite^(^ ^11^ ^)^, com arguição sobre ocorrência, frequência e intensidade de espirros e coriza, além do diagnóstico médico prévio da doença. Consideraram-se hígidas as crianças cuja pontuação do módulo de asma esteve ≤5 e, no módulo rinite, ≤4. Também se aplicou um recordatório de saúde da criança, elaborado pelos pesquisadores, constando questões relativas à prática de atividade física, uso de medicamentos, doenças diagnosticadas vigentes ou pretéritas e internações hospitalares, para confirmar a higidez. 

Excluíram-se os escolares com história de doença cardiorrespiratória, neuromuscular, reumatológica ou neurológica e aqueles com qualquer doença aguda no momento da coleta ou impossibilidade de realização adequada de algum dos procedimentos de avaliação. Além desses, escolares cujo questionário de saúde tivesse respostas duvidosas quanto à higidez da criança não compuseram a amostra, bem como aqueles com volume expiratório forçado no primeiro segundo (VEF_1_) menor que 80% do predito, segundo Polgar e Weng^(^ ^12^ ^)^. 

Após consentimento das escolas quanto à participação, conduziu-se a coleta na instituição de ensino, sempre pelo mesmo avaliador, em um espaço reservado. Todos os participantes receberam e devolveram o termo de consentimento livre e esclarecido assinados pelos pais ou responsáveis legais. O estudo foi aprovado pelo Comitê de Ética e Pesquisa em Seres Humanos da Universidade do Estado de Santa Catarina. 

Inicialmente, avaliaram-se peso corporal (precisão de 0,1kg) e estatura (precisão de 0,5cm). A obtenção dessas medidas antropométricas foi conduzida com a criança permanecendo com o corpo ereto e alinhado, tendo calcanhares, panturrilhas, glúteos, escápulas e região do occipital tocando o estadiômetro. No momento da avaliação, os participantes vestiam bermuda ou calça do uniforme escolar e camiseta e estavam descalços. Posteriormente, os valores previamente obtidos de 150g para camiseta, 150g para bermuda ou 250g para calça foram subtraídos do valor de peso aferido.

Conhecidos os valores de peso corporal e estatura, calculou-se o índice de massa corpórea (IMC) por meio da calculadora de IMC infantil do Ministério da Saúde^(^ ^13^ ^)^. Trata-se de um instrumento que dispõe de campos onde se inserem os dados de peso, estatura, sexo e idade da criança. Uma vez processados, obtém-se o valor do IMC, o percentil e, a partir deste, o diagnóstico de trofismo. Com base nessas informações, reuniram-se as crianças em três grupos, determinados pelo percentil em que a criança se encontrava na curva IMC *versus* idade, a saber: Grupo Eutrófico (GE - para os pertencentes aos percentis maiores que 3 e menores que 85); Grupo Sobrepeso (GS - para os percentis iguais ou maiores que 85 e iguais ou menores que 97); e Grupo Obeso (GO - quando os percentis eram maiores que 97)^(^ ^13^ ^)^.

Após a antropometria, o mesmo avaliador realizou a mensuração do VEF_1_, aferido com um monitor digital (PIKo-1, Spire Health, EUA). As medidas foram tomadas seguindo-se as normas e critérios da declaração para função muscular respiratória da *American Thoracic Society* (ATS) e da *European Respiratory Society* (ERS)^(^ ^14^ ^)^ com a criança sentada, as costas apoiadas no encosto da cadeira, a cabeça alinhada e os membros superiores repousados sobre os inferiores. Utilizou-se um clipe nasal e a criança foi orientada a realizar uma inspiração máxima seguida de expiração forçada, com estímulo verbal. Registrou-se o maior valor de três medidas com intervalo de 30 segundos entre elas, sendo que duas não deveriam diferir mais do que 0,15L, em um número máximo de cinco manobras. Em não se obtendo medidas aceitáveis, desconsiderou-se o teste.

Em seguida, verificou-se a FMR por meio de um manovacuômetro digital com válvula unidirecional (MVD300, G-MED, Brasil). O sistema de aferição apresenta um orifício de 2mm de diâmetro para evitar fechamento glótico durante a manobra de pressão inspiratória máxima (PIMáx) e reduzir o uso de músculos bucais durante a manobra de pressão expiratória máxima (PEMáx). Após orientações e demonstrações sobre o teste, o avaliador ofereceu incentivo verbal para que o escolar realizasse uma inspiração máxima, seguida de expiração máxima, através de um bocal mantido firmemente ao redor dos lábios para evitar vazamentos. Durante a execução do teste, o escolar permaneceu sentado, com as costas no encosto da cadeira, pés apoiados no chão, membros superiores repousando sobre os inferiores e fez uso de um clipe nasal. Realizaram-se as medidas segundo as normas e critérios da declaração para função muscular respiratória da ATS^(^ ^15^ ^)^. Para se obter a PIMáx, a criança expirou até o próximo do volume residual e, em seguida, realizou uma inspiração máxima. A PEMáx foi mensurada a partir de uma inspiração próxima da capacidade pulmonar total, seguida de uma expiração máxima. Realizaram-se no mínimo três e no máximo sete manobras para cada uma das medidas de PIMáx e PEMáx. Não sendo obtidas medidas aceitáveis e reprodutíveis, o teste foi considerado inválido. Consideraram-se as medidas satisfatórias quando o valor máximo de três manobras aceitáveis - sem vazamentos e com duração de pelo menos 2 segundos - e reprodutíveis variaram menos de 20% entre si, sendo registrada a maior medida. A cada manobra de cada medida, houve um intervalo de 30 a 40 segundos^(^ ^15^ ^)^. Entre a aferição da PIMáx e da PEMáx, houve um intervalo de 3 minutos, para evitar o cansaço do escolar avaliado. 

Para o cálculo do tamanho amostral, considerou-se o resultado da PIMáx de um estudo-piloto, no qual a mesma apresentou um desvio padrão de 10cmH_2_O. Para detectar uma diferença de 5cmH_2_O e um poder do teste de 80%, com nível de significância de 5%, estimaram-se 25 escolares em cada um dos grupos^(^ ^16^ ^)^. 

Para análise dos dados, os parâmetros numéricos foram importados para o Microsoft Excel^(r)^ 2010 e, na sequência, transferidos ao *software Statistical Package for the Social Sciences* (SPSS) 20.0 *for Windows* para o processamento estatístico. Inicialmente, empregou-se estatística descritiva e de frequências, sendo os dados expressos em médias e desvio padrão. Aplicou-se o teste de normalidade de Kolmogorov-Smirnov e, posteriormente, para comparar os três grupos, utilizou-se o teste não paramétrico de Kruskal-Wallis. Constatada a diferença entre os grupos, aplicou-se o teste de Mann-Whitney para buscar as diferenças comparando-se dois grupos por vez. Para identificar as correlações entre as variáveis, aplicou-se a correlação de Spearman. O nível de significância considerado foi de 0,05.

## Resultados

Somando as três instituições envolvidas, avaliaram-se 112 escolares e 90 fizeram parte da amostra, sendo 30 em cada um dos grupos, com 15 de cada sexo e idades de sete a nove anos. Do total de crianças avaliadas, excluíram-se 16 devido a doença crônica ou aguda vigente, quatro por apresentarem VEF_1_ menor que 80% do predito e dois por não concluírem os testes solicitados. A caracterização da amostra de cada um dos três grupos, segundo os dados antropométricos de peso, estatura, IMC e percentil, e as variáveis respiratórias PIMáx, PEMáx e VEF_1_ são apresentadas na Tabela 1. 

**Tabela 1:** Caracterização da amostra segundo as variáveis antropométricas e respiratórias

Como já esperado diante da prévia caracterização dos grupos em GE, GS e GO pelo IMC, constatou-se diferença significativa nas variáveis antropométricas de peso (*p*<0,001), IMC (*p*<0,001) e percentil (*p*<0,001), na comparação entre os grupos por meio do teste de Kruskal-Wallis. Das variáveis respiratórias, os grupos diferiram apenas na PIMáx (*p*=0,033). A análise entre dois grupos de cada vez por meio do teste de Mann-Whitney identificou diferença na PIMáx, com maiores valores para o GE comparado aos outros grupos (GE - 63,9±9,75 *versus* GS - 58,6±13,81, *p*=0,043; GE *versus* GO - 58,36±16,14, *p*=0,013), sem diferença significante entre GS e GO (58,6±13,81 *versus* 58,36±16, *p*=0,779). Não houve diferença entre as pressões respiratórias máximas (PRM), segundo o sexo, em nenhum dos três grupos. 

Aplicada a correlação de Spearman entre os dados das variáveis da amostra total, observou-se que a idade correlacionou-se com as variáveis de VEF_1_ (r=0,578), PIMáx (r=0,281) e PEMáx (r=0,328). Também houve correlação negativa da PIMáx com o percentil e a classificação segundo o IMC (IMCclass) (r=-0,214 e r=-0,256). As PRM se correlacionaram fortemente entre si (r=0,822) e em menor intensidade com o VEF_1_ (PIMáx, r=0,362 e PEMáx, r=0,494). A PEMáx apresentou relação com a estatura (r=0,328) e com o VEF_1_ (r=0,488).

Na análise de cada um dos grupos, a idade apresentou valores de correlação, exceto no GS, com o peso (GE - r=0,722; GO - r=0,380) e estatura (GE - r=0,772; GO - r=0,513). O peso correlacionou-se também, como esperado, com a altura (GE - r=0,895; GS - r=0,910; GO - r=0,843) e com o IMC (GE - r=0,848; GS - r=0,530; GO - r=0,896). A PIMáx guardou correlação com a idade somente no GE (r=0,389), com o VEF_1_ no GO (r=0,565) e com a PEMáx em todos os grupos (GE - r=0,773; GS - r=0,795; GO - r=0,910). A PEMáx também correlacionou-se com a idade e a estatura no GE (r=0,413 e r=0,479, respectivamente) e com o VEF_1_ nos três grupos (GE - r=0,531; GS - 0,429; GO - r=0,569). O VEF_1_, por sua vez, correlacionou-se com a idade (GE - r=0,541; GS - r=0,663; GO - r=0,438), o peso (GE - r=0,438; GS - r=0,461) e a estatura (GE - r=0,515; GS - r=0,379).

## Discussão

A musculatura respiratória é a responsável por gerar as diferenças de pressão que asseguram a ventilação pulmonar e, por isso, a medida da FMR é considerada indispensável e de grande utilidade na avaliação de vários estados e enfermidades^(^ ^17^ ^)^. Entre essas situações clínicas, a preocupação com a obesidade infantil tem sido crescente. No entanto, as implicações da obesidade sobre a FMR ainda não estão bem definidas, o que motivou a presente investigação.

Dentre os resultados obtidos, identificou-se correlação entre a idade e as PRM, tanto na amostra total quanto no GE, o que se encontra em consonância com outros estudos^(^ ^18^ ^-^ ^21^ ^)^. Interessante observar que o evento não se repetiu no GS e no GO, o que pode ser um indicativo do comprometimento das PRM na presença de alteração no trofismo corporal, no caso, do excesso de peso. Tal observação pode ser explicada pelas alterações provocadas pelo acúmulo de gordura nas cavidades torácica e abdominal, o que pode traduzir-se em prejuízo à bomba ventilatória pela alteração funcional da musculatura inspiratória e expiratória^(^ ^5^ ^-^ ^8^ ^)^.

A idade também se correlacionou com o VEF_1_ em todas as análises, sendo que essa variável espirométrica demonstrou estreita relação com a estatura. Tal correspondência é justificada pela proporcionalidade do crescimento corporal e respiratório na infância, que acontecem com o avanço da idade^(^ ^22^ ^)^. A literatura já tem descrito esse evento^(^ ^12^ ^,^ ^23^ ^-^ ^25^ ^)^, no qual o ganho de estatura ocorre em função da idade e a função pulmonar é caracterizada pelo aumento dos volumes acompanhando o crescimento durante a infância e adolescência. 

Essa linearidade no desenvolvimento somático também pode fundamentar a forte correlação verificada entre PIMáx e PEMáx. No caso dessa relação entre as PRM, outro ponto que sustenta a correlação é a contiguidade anatômica e funcional entre os compartimentos torácico e abdominal. O ato inspiratório, que, *a priori*, ocorre no compartimento torácico, se dá de maneira mais efetiva quando o diafragma encontra uma musculatura abdominal suficientemente forte para lhe dar sustentação no movimento, efetivando o sinergismo muscular. Na amostra composta por crianças hígidas, parece ser adequada a observação de que as pressões crescem de forma conjunta com a idade, proporcionando à criança respostas inspiratórias e expiratórias apropriadas nas situações de aumento de demanda ventilatória^(^ ^26^ ^)^.

Quanto à PIMáx, verificou-se decréscimo nos valores de GE, GS e GO, corroborada pelas correlações negativas - mesmo que fracas - da variável com o percentil e também com a classificação segundo o IMC. Esse achado pode ser explicado por uma disfunção do diafragma relacionada à deposição de tecido adiposo abdominal e visceral, que levaria a uma desvantagem na relação comprimento-tensão, devido ao estiramento excessivo das fibras musculares. Os efeitos ocorrem principalmente na musculatura inspiratória, sobretudo no diafragma, sendo o prejuízo à função pulmonar agravado conforme o grau de obesidade^(^ ^9^ ^,^ ^27^ ^,^ ^28^ ^)^. Tais argumentos poderiam justificar também a correlação negativa encontrada entre a PIMáx e as variáveis percentil e classificação do trofismo segundo o IMC, caracterizando o maior grau de sobrepeso/obesidade com maior tendência a prejuízo da FMR. Corroborando essa linha, Santiago *et al**^(^* ^8^ ^)^ avaliaram crianças e adolescente agrupados em eutróficos e sobrepeso/obesos de quatro a 15 anos e constataram maiores valores de PEMáx no grupo eutrófico (*p*=0,003). Os pesquisadores discutem uma tendência à diminuição da PIMáx para o grupo sobrepeso/obeso (*p*=0,068), atribuindo o achado à distribuição abdominal da adiposidade e aos seus efeitos sobre as PRM. 

Ainda comparando a FMR de obesos e eutróficos, um estudo tailandês^(^ ^29^ ^)^ não encontrou diferenças significantes entre os grupos, na avaliação de crianças de dez a 12 anos. Para Charususin *et al**^(^* ^29^ ^)^, a adiposidade não interferiu nos resultados da amostra, uma vez que os participantes apresentavam valores de função pulmonar dentro do predito. Outro trabalho^(^ ^9^ ^)^, que incluiu crianças maiores e adolescentes (de nove a 17 anos), não evidenciou influência do peso corporal sobre as PRM. Segundo os autores, o achado pode ser decorrente do efeito do constante treinamento exercido pela sobrecarga inspiratória imposta pelo acúmulo do tecido adiposo.

Diferentemente deste estudo, os trabalhos mencionados, que avaliaram a FMR segundo o trofismo, analisaram conjuntamente crianças e adolescentes, o que pode ter influenciado nos resultados obtidos. Isso porque são notáveis as modificações corporais e ventilatórias a partir da transição dessas duas etapas da vida^(^ ^12^ ^,^ ^24^ ^,^ ^26^ ^)^. Não se encontraram estudos com metodologia semelhante à da presente investigação em crianças de sete a nove anos de idade, impedindo comparações entre os resultados. No que se refere aos valores absolutos de PIMáx e PEMáx, quando tomadas somente as crianças do GE, observa-se que os valores descritos na literatura são maiores que os identificados no presente estudo. Domènech-Clar *et al**^(^* ^20^ ^)^ encontraram, em sua amostra, PIMáx de 79 e 68cmH_2_O e PEMáx de 95 e 82cmH_2_O para meninos e meninas de oito a dez anos, respectivamente. Nessa mesma linha, valores maiores são apresentados por Wilson^(^ ^18^ ^)^ e Szeinberg^(^ ^19^ ^)^, ambos também referenciados pela ATS^(^ ^15^ ^)^. Essa mesma publicação^(^ ^15^ ^)^ chama a atenção para a necessidade da regionalização dos valores de referência e consequente cuidado na extrapolação da interpretação dos resultados.

Como discutido, o impacto da obesidade sobre a função ventilatória tem sido frequentemente discutido, evidenciando-se, na presente investigação, seus efeitos especialmente sobre a PIMáx. Considerando o aumento de peso das crianças como um fenômeno de Saúde Pública em contínua expansão, os achados do estudo apontam para a relevância da monitorização da FMR, principalmente nas crianças com maiores índices de massa corpórea, para prevenir possíveis distúrbios respiratórios e seus agravos. Nesse contexto, é importante destacar uma limitação da presente pesquisa quanto à inclusão dos participantes. O equipamento utilizado para a avaliação respiratória dos escolares, o PIKo-1 (Spire Health, EUA), não fornece o dado de capacidade vital forçada (CVF), inviabilizando a análise da relação VEF_1_/CVF para excluir apenas as crianças com doença respiratória de caráter obstrutivo.

Pode-se concluir que escolares obesos e com sobrepeso apresentaram valores inferiores de PIMáx, em comparação aos eutróficos. Houve forte relação entre PIMáx e PEMáx, tendo ambas relação com idade e VEF_1_, principalmente no GO. A PIMáx se relacionou com o IMC e a PEMáx, com a estatura, especialmente no GE. Os achados apontam para a influência de variáveis antropométricas na FMR em crianças, bem como para a relação entre a força e o parâmetro espirométrico de VEF_1_.
